# Dynamic brain communication underwriting face pareidolia

**DOI:** 10.1073/pnas.2401196121

**Published:** 2024-04-08

**Authors:** Valentina Romagnano, Julian Kubon, Alexander N. Sokolov, Andreas J. Fallgatter, Christoph Braun, Marina A. Pavlova

**Affiliations:** ^a^Social Neuroscience Unit, Department of Psychiatry and Psychotherapy, Tübingen Center for Mental Health, Medical School and University Hospital, Eberhard Karls University of Tübingen, Tübingen 72076, Germany; ^b^Magnetoencephalography Center, Medical School and University Hospital, Eberhard Karls University of Tübingen, Tübingen 72076, Germany; ^c^Hertie Institute for Clinical Brain Research, Medical School and University Hospital, Eberhard Karls University of Tübingen, Tübingen 72076, Germany

**Keywords:** face pareidolia, social cognition, magnetoencephalography, brain communication, gamma oscillations

## Abstract

Face pareidolia is a tendency to seeing faces in nonface images that reflects high tuning to a face scheme. Yet, studies of the brain networks underwriting face pareidolia are scarce. Here, we examined the time course and dynamic topography of gamma oscillatory neuromagnetic activity while administering a task with nonface images resembling a face. Images were presented either with canonical orientation or with display inversion that heavily impedes face pareidolia. At early processing stages, the peaks in gamma activity (40 to 45 Hz) to images either triggering or not face pareidolia originate mainly from the right medioventral and lateral occipital cortices, rostral and caudal cuneus gyri, and medial superior occipital gyrus. Yet, the difference occurred at later processing stages in the high-frequency range of 80 to 85 Hz over a set of the areas constituting the social brain. The findings speak rather for a relatively late neural network playing a key role in face pareidolia. Strikingly, a cutting-edge analysis of brain connectivity unfolding over time reveals mutual feedforward and feedback intra- and interhemispheric communication not only within the social brain but also within the extended large-scale network of down- and upstream regions. In particular, the superior temporal sulcus and insula strongly engage in communication with other brain regions either as signal transmitters or recipients throughout the whole processing of face-pareidolia images.

## Significance

Face-pareidolia images represent a valuable tool for studying face tuning as their single elements do not trigger face processing. What happens in the brain during face pareidolia? The present analysis indicates that mutual feedforward and feedback communication unfolds over time not only within the social brain but also within the extended large-scale network of down- and upstream regions serving either as signal transmitters or recipients. Assuming alterations in dynamic brain communication account for social cognition deficits, the outcome provides a framework for research in patients with mental and neurological conditions such as autism spectrum disorders, schizophrenia, and Parkinson’s disease.

Face pareidolia is a tendency to see faces in nonface images such as grilled toasts, landscapes, or still-life fruits (1–14). This ability implies high tuning or a kind of predisposition to a coarse face scheme such *as two eyes above a mouth* in the visual input (10, 15). Such a rough face scheme (or template) is considered to emerge early in the lifespan or even be hardwired in the brains of humans, nonhuman primates, domestic chicks, and even in species without parental care (16–20). Although face pareidolia does not represent a uniquely human phenomenon, it appears to be more pronounced in humans. Preschool children, first trained to select faces among nonface images, later choose not only faces but also face-like nonfaces, whereas rhesus monkeys (*Macaca mulatta*) and capuchin monkeys (*Sapajus apella*) do not (21).

Face-pareidolia images represent a valuable tool for studying face sensitivity as they do not contain specific facial cues that implicate face presence. Behavioral studies point to a shared mechanism for emotion recognition between faces and face-like images suggesting that this information is not limited to faces (22). Moreover, face-like nonfaces convey information not only about emotional expressions but also personal characteristics and traits such as trustworthiness and dominance, and even age and gender (2, 13, 23).

What happens in the brain during face pareidolia? Is processing of face-like images similar to that of faces? Up to date, studies on the brain networks underwriting face pareidolia are sparse and the outcome is controversial. A great portion of neuroimaging research was conducted with portraits of Giuseppe Arcimboldo, a genius Italian painter (1526 to 1593), whose masterpieces represent faces as compositions of fruits or plants (24–27). Functional MRI (fMRI) shows that Arcimboldo portraits elicit right-hemispheric activation of the essential nodes of the social brain engaged in face processing such as the fusiform face area (FFA), posterior superior temporal sulcus (STS), and inferior frontal gyrus (24, 26). Electroencephalography (EEG) indicates that the amplitude of N170 associated with face processing, is similar for both Arcimboldo portraits and faces in the right (but not left) occipitotemporal region (25). No difference occurs in the amplitude of P1, N170, and N250 for faces and Arcimboldo paintings, whereas with display inversion, the N170 amplitude does not segregate Arcimboldo paintings from objects (27).

Another portion of brain imaging deals with naturalistic Face-n-Thing images of objects and scenes resembling a face. Whole-brain fMRI and source localization of EEG activity indicate that both faces and Face-n-Thing images elicit activation of similar topography in the face-selective temporal areas such as the FFA and prefrontal regions (5, 28). Increased fMRI activation to the Face-n-Thing as compared with nonface images occurs in the occipital cortex, precentral gyrus, and insula of both hemispheres as well as in the right thalamus and cerebellum (29). Brain processing of faces and Face-n-Thing images is different already at early stages: The N170 amplitude is larger and latency shorter for faces (5, 30, 31). Conversely, the evoked magnetoencephalographic (MEG) M165 response over the right ventral FFA to faces and Face-n-Thing images is rather similar: The brain appears to be hardwired to detect a face (32). In accord with this, the face-specific regions in the right hemisphere are reported to be active in response to Face-n-Thing images at 160 ms from stimulus onset, whereas later, at 260 ms, face-like images are processed similarly to nonface objects (12).

Taken together, the data indicate that topography of the neural circuits underwriting processing of faces and face-like images is rather similar, with predominant right-hemispheric lateralization. It is unclear, however, i) which neural networks underwrite processing of face-like images; ii) whether face-like nonfaces eliciting and not eliciting face pareidolia are treated in similar ways already at early or rather late processing stages, and iii) how the key brain areas underwriting face pareidolia communicate with each other in time.

Face pareidolia is likely to be supported by gamma oscillatory brain activity, which is commonly believed to underlie the perception of *Gestalt*. Gamma oscillations [above 30 Hz; (33)] are linked not only to a wide range of cognitive abilities such as working memory and selective attention (34, 35) but also underpin processing of social signals such as faces, Mooney faces, point-light body motion, and social interaction in Heider-and-Simmel-like animations (36–41). Alterations in the gamma MEG response to Arcimboldo-like images triggering face pareidolia occur both in typically developing individuals and patients with major depressive disorder [MDD; (8)].

**Fig. 1.**
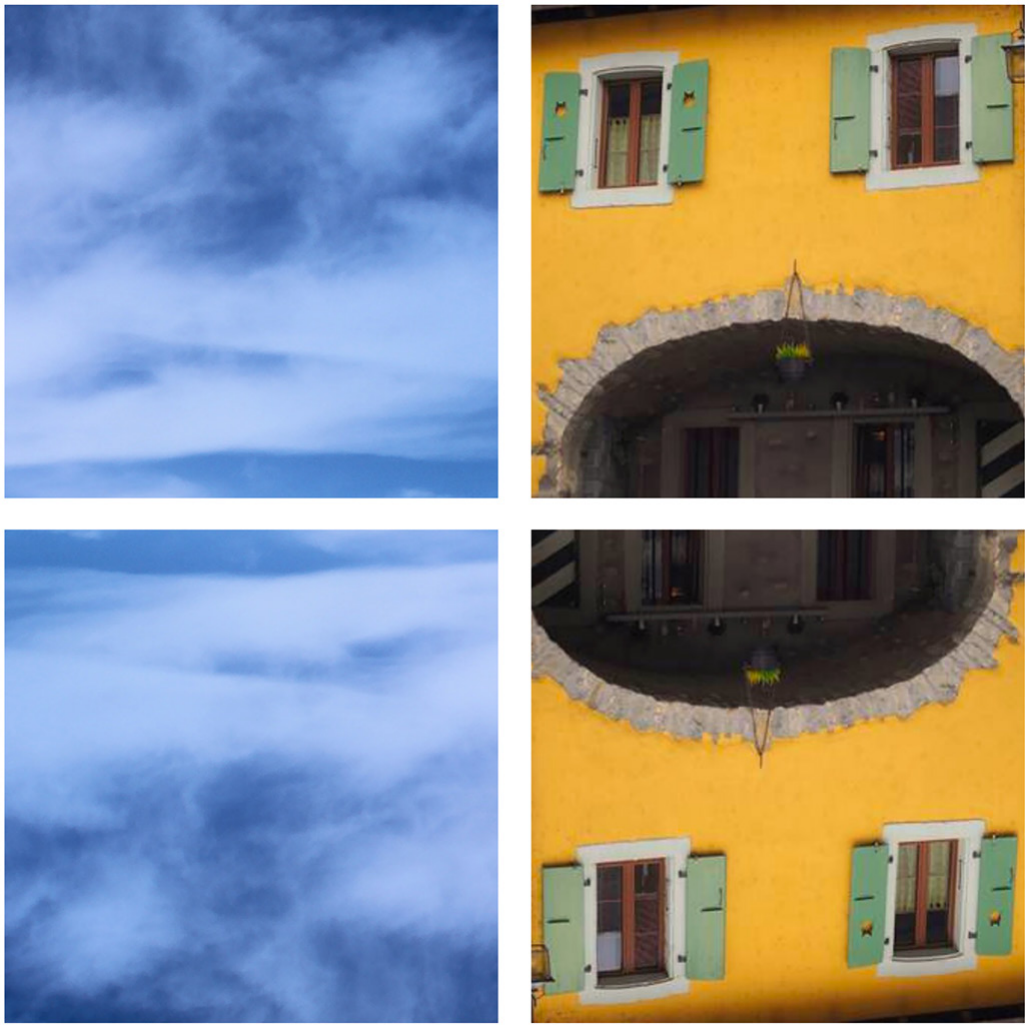
Examples of the Face-n-Thing images with canonical upright (*Top*) and inverted (*Bottom*) display orientation. The image on the *Left* is one of the least resembling a face with upright display orientation, and the image on the *Right* is one of the most resembling a face when presented with upright orientation. Image credit: The right panel reprinted from ref. 3, the left panel from ref. 11, both of which are licensed under CC BY 4.0.

The present work is directed at revealing neural circuits underwriting face pareidolia and, in particular, brain communication unfolding over time. With this purpose in mind, we used MEG, a noninvasive brain imaging technique providing for high temporal resolution. We analyzed gamma oscillatory activity during processing of Face-n-Thing images (3, 10, 11) either with canonical orientation or display inversion that severely impedes face pareidolia (Fig. 1). Display inversion provides a proper control for face tuning since the overall amount of intrastimulus information is the same with both orientations. On each trial, in a two-alternative forced-choice (2AFC) task, participants had to indicate whether or not they had an impression of a face.

## Results

### Behavioral Data Analysis.

As expected from previous behavioral work (3, 10, 11), the impact of display inversion on face response rate was highly significant (face pareidolia with upright orientation, mean ± SD, 0.576 ± 0.171; median (Mdn), 0.612, 95% CI [0.500 to 0.652]; with inversion, 0.363 ± 0.241, Mdn, 0.330, 95% CI [0.256 to 0.470]; Wilcoxon signed-rank test, *z* = 3.66, *P* = 0.0003, two-tailed; effect size, eta squared *η*^2^ = 0.61). The difference in face response rate with upright orientation and no-face response rate with display inversion (0.628 ± 0.243, Mdn, 0.656, 95% CI [0.520 to 0.736]) was nonsignificant (Wilcoxon signed-rank test, *z* = 0.70, *P* = 0.487, two-tailed; n.s.).

### MEG Analysis Outcome.

#### Increases in gamma oscillations.

**Fig. 2.**
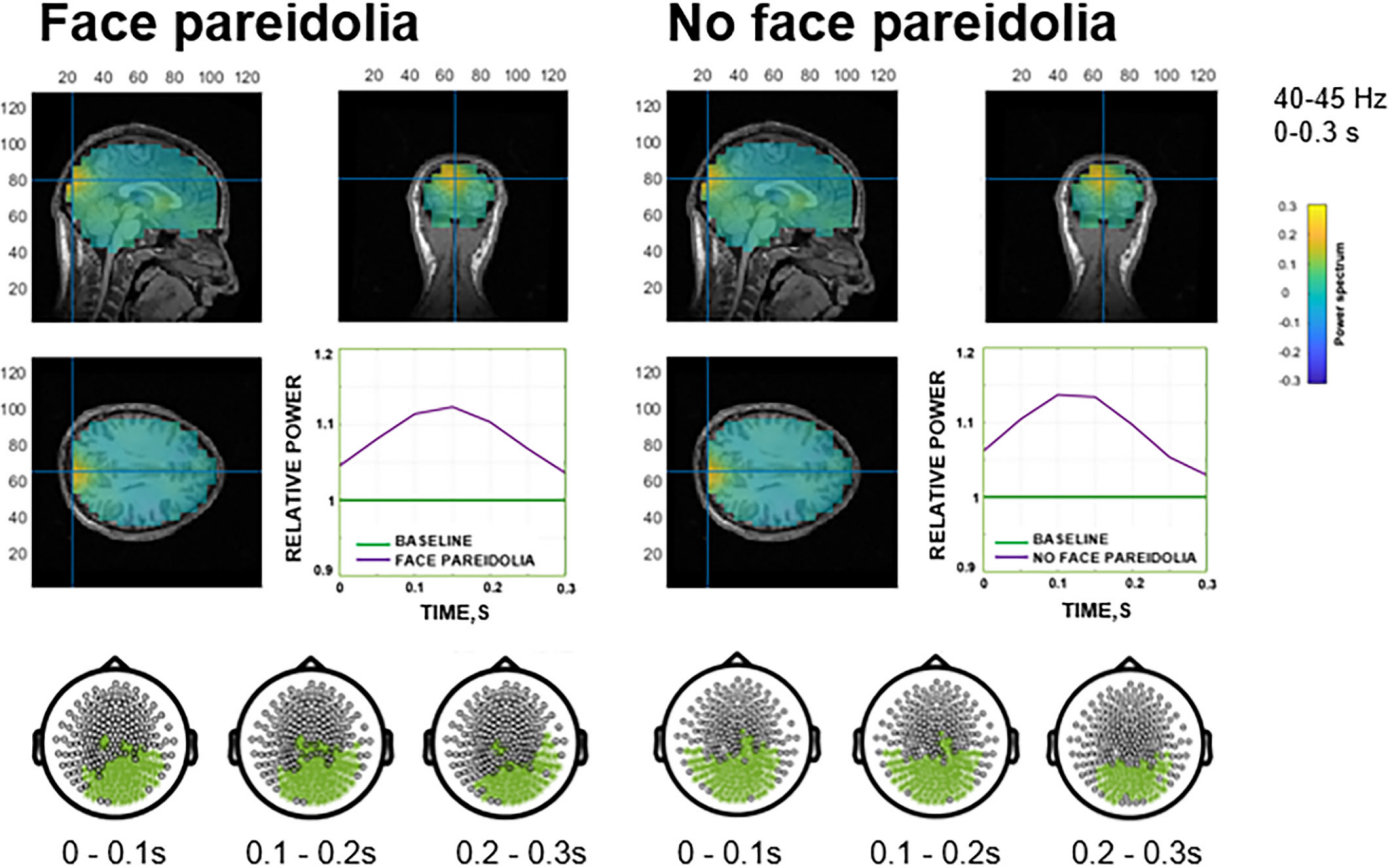
Visualization of significant clusters and source reconstruction separately for the *face pareidolia* (*Left*) and *no face pareidolia* (*Right*) responses versus baseline. Increases in gamma oscillatory activity (40 to 45 Hz) during early processing (0 to 300 ms) originated primarily from the right MVOC. In the source plots, greater activity relative to baseline is represented in yellow. Channels constituting the significant positive clusters (*Bottom* rows of each panel) are highlighted in green. The time course covering the first 300 ms from stimulus onset (*Bottom Right* corner of each panel) displays the relative power for each condition (*face pareidolia* or *no face pareidolia*, violet) compared to baseline (green).

For the face responses, significant increases in gamma activity compared with baseline (*P* = 0.003, corrected throughout for multiplicity) occurred in the frequency range of 40 to 45 Hz during the whole stimulus duration (0 to 1.2 s). For the no-face responses, a significant increase was observed in the early gamma response (from 0 to 0.3 s) in the frequency ranges of 35 to 40 Hz (*P* = 0.03) and 40 to 45 Hz (*P* = 0.004). As early gamma activity peaked for both the face and no-face responses, these early increases were not stimulus-specific. As seen from Fig. 2, source localization analysis indicated that these bursts originated from the areas heavily involved in the visual processing with the maximum in the medioventral occipital cortex (MVOC) of the right hemisphere.

#### Face versus no-face contrast.

**Fig. 3.**
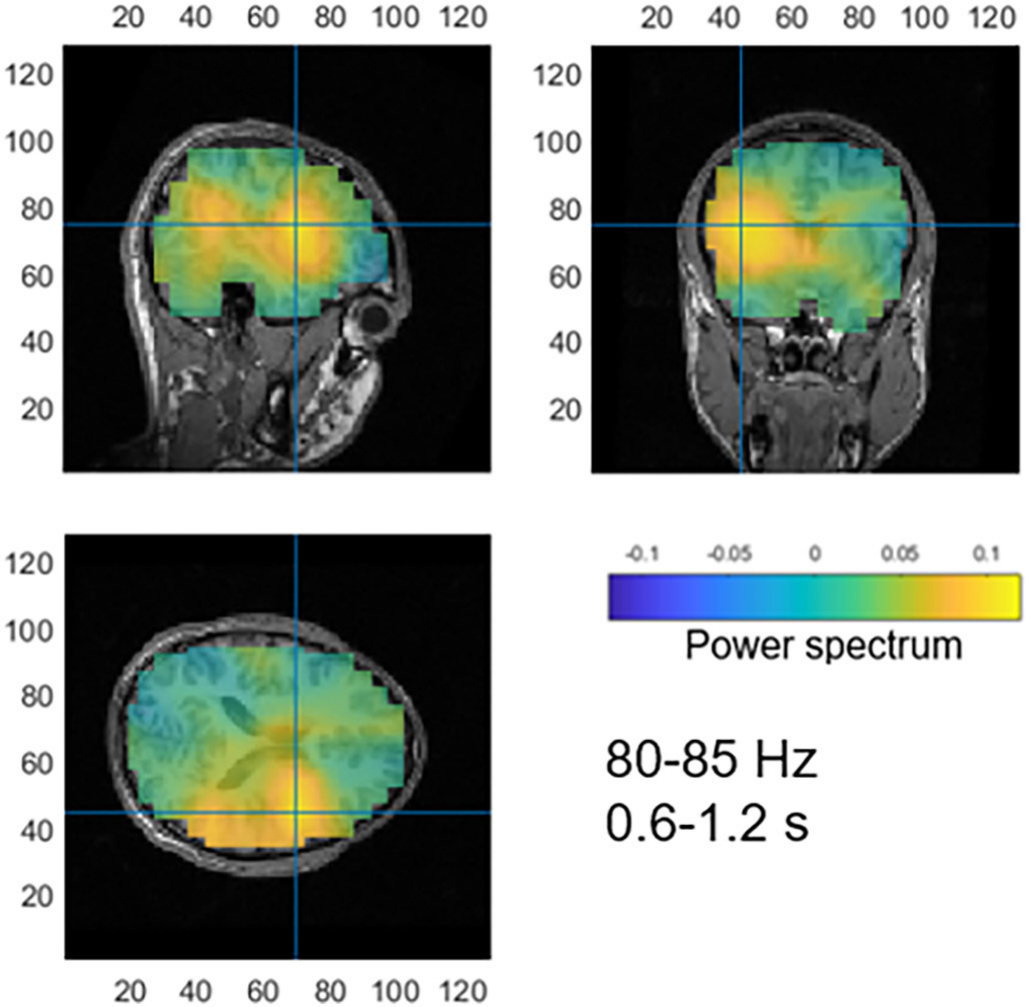
Source reconstruction for the contrast between images eliciting face pareidolia and not. The greatest peak in gamma activity (80 to 85 Hz) was found in the INS-R (marked with a blue cross).

Contrast of gamma activity in response to images triggering face pareidolia (face response) with images that did not elicit face pareidolia (no-face response) resulted in several significant clusters of gamma oscillations in the range of 80 to 85 Hz at later latencies only (from 0.6 to 1.2 s, *P* = 0.003). The source localization analysis uncovered multiple clusters of increased gamma activity in the right posterior STS (STS-R), right inferior temporal gyrus (ITG-R), right insular gyrus (INS-R), right postcentral gyrus (PoG-R), and the left inferior parietal lobule (IPL-L). The greatest peak was found in the INS-R (Fig. 3).

#### Time-based connectivity analysis.

**Fig. 4.**
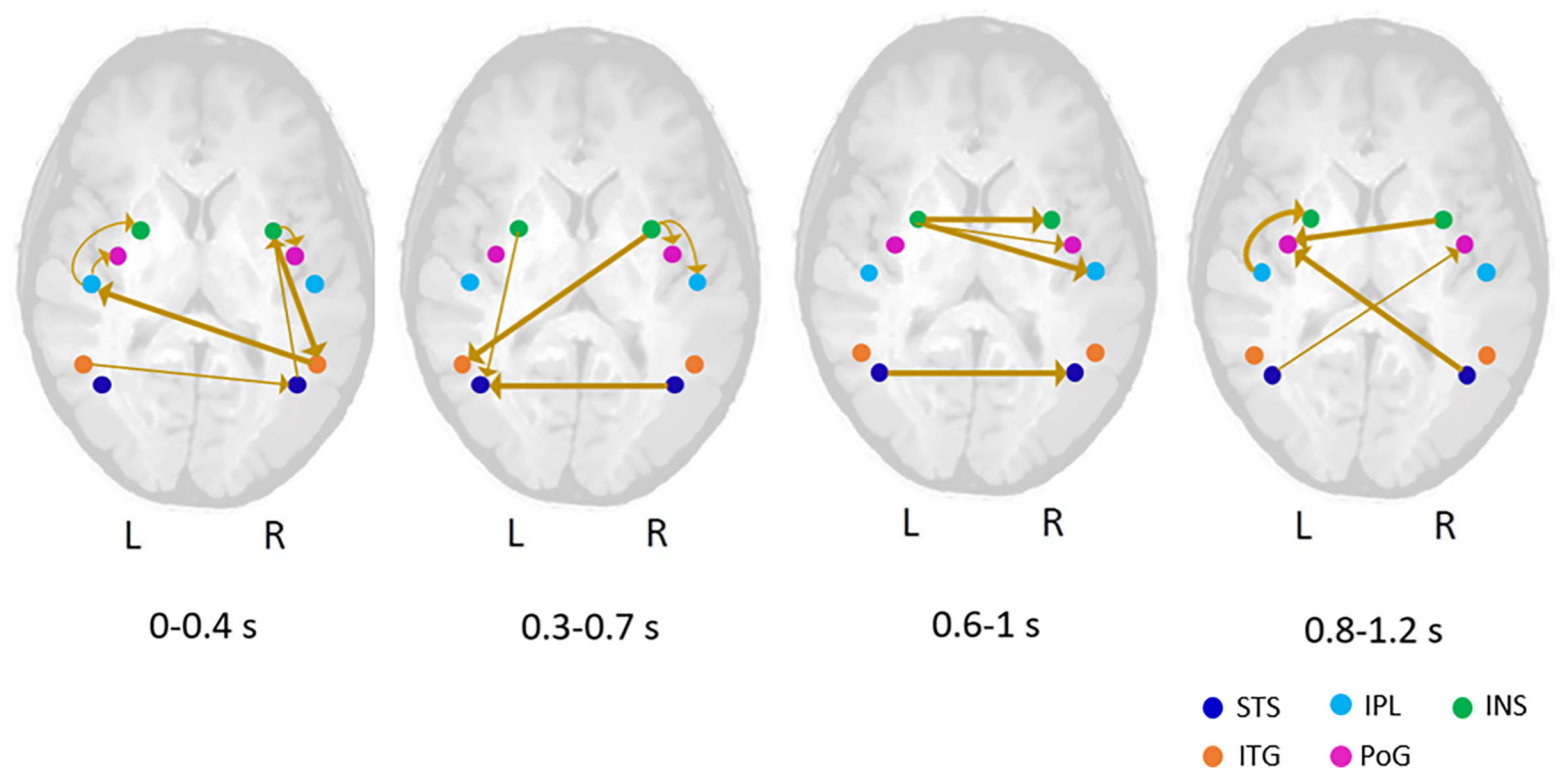
Functional connectivity unfolding over time between the brain regions with increased gamma oscillatory MEG activity for images triggering face pareidolia. Significant connections superimposed on the schematic brain are shown by thick arrows (*P* < 0.05), while thin arrows represent connections that tend to be significant (*P* < 0.08). Colored blobs indicate the regions of interest: STS—superior temporal sulcus, IPL—inferior parietal lobule, INS—insula, ITG—inferior temporal gyrus, and PoG—postcentral gyrus.

Fig. 4 represents the outcome of the connectivity analysis unfolding over time between the regions with increased gamma activity for images eliciting face pareidolia. The detailed description is given in *SI Appendix, Supplementary Material*. As seen from Fig. 4, mutual feedforward and feedback (both intra- and interhemispheric) communication occurs not only within the social brain but also beyond, within the extended large-scale network of down- and upstream regions. The key areas of the social brain, in particular, the STS and INS in both brain hemispheres, play a decisive role (like two kingpins) being strongly engaged in communication with other brain regions either as signal transmitters or recipients during the whole processing of face-pareidolic images. In the first time window (0 to 0.4 s), the STS-R receives messages from the ITG-L and transfers information to the INS-R. Later on (0.3 to 0.7 s), the STS-L receives signals from the STS-R and INS-L, then (0.6 to 1 s) the STS-L transmits signals to the STS-R, and finally (0.8 to 1.2 s), both the right and left STS transmit signals to the PoG of the opposite hemisphere. Similarly, the left and right INS are never silent. In the first time window, the INS-R receives signals from the STS-R and transfers information to the ITG-R and PoG-R, whereas the INS-L receives signals from the IPL-L. Later on, both the INS-L and INS-R transmit signals to the STS-L and ITG-L, respectively. Then, the INS-L transmits information to the INS, PoG, and IPL of the opposite hemisphere, and finally, the INS-R transfers information to the PoG-L, while the INS-L receives information from the IPL-L.

## Discussion

This work was aimed at investigation of brain circuits underpinning face pareidolia. For this purpose, we administered a face pareidolia task with a recently created set of naturalistic Face-n-Thing images (3, 10, 11) while recording MEG activity. The outcome indicates: i) At early processing stages, for images either triggering face pareidolia or not, the bursts of gamma oscillatory activity (40 to 45 Hz) are rather similar. In terms of topography, the peaks originate mainly from the areas heavily involved in visual processing of scenes and objects such as the right MVOC and lateral occipital cortex, rostral and caudal cuneus gyri, and the medial superior occipital gyrus. ii) The difference in processing of images eliciting and not eliciting face pareidolia occurs at later processing stages in the high-frequency gamma range of 80 to 85 Hz over a number of areas constituting the social brain such as the STS and INS. The findings, therefore, speak for a relatively late neural network playing a decisive role in face pareidolia. iii) Most strikingly, a cutting-edge analysis of brain connectivity unfolding over time uncovers mutual feedforward and feedback communication not only within the social brain but also within the extended large-scale network of down- and upstream regions. In particular, the STS and INS serve as key players engaged in brain communication either as signal transmitters or recipients throughout the whole processing of face-pareidolia images.

### Face Pareidolia: Early or Late Processing?

The present study indicates the lack of difference in early processing of Face-n-Thing images either triggering or not face pareidolia. The peaks of gamma oscillatory activity (40 to 45 Hz) of similar topography originate mainly from the areas profoundly involved in visual processing of scenes and objects. Noteworthily, gamma power exhibits positive correlations and high topographical correspondence with the hemodynamic blood oxygenation-level dependent signal in fMRI studies (42).

At first glance, this outcome contradicts some previous findings suggesting early alterations in brain activity in response to face-pareidolia images. However, most of preceding research compared the brain response to face-pareidolia images with faces in favor of more efficient processing of a face. For instance, EEG indicates brain processing of faces and face-like images is different already at earlier stages: The amplitude of N170 (150 to 210 ms) in the fusiform gyrus and ITG is larger and latency shorter for faces (5, 30, 31). Similarly, latency of the vertex positive potential (VPP; 150 to 190 ms) is shorter, and both the VPP and N250 amplitudes are larger for faces (5). On the other hand, at 160 ms, the right-hemispheric face-specific regions reportedly exhibit comparable responsiveness to both face-like images and faces, while later, at 260 ms, face-like images are processed more similar to nonfaces (12). The amplitude of M165 in the FFA is similar for faces and face-like images but is different for objects (32). Recent single-unit recording indicates that at short latencies, the majority of face-selective neurons in the human right inferior occipital gyrus (the occipital face area) are responsive not only to faces but also to feature-scrambled faces and Face-n-Thing images (43). The present study suggests the specificity of the gamma oscillatory MEG response to images triggering face pareidolia later in time (0.6 to 1.2 s) rather than at early processing stages.

Several factors may be responsible for the lack of difference in early MEG gamma activity between images either eliciting or not face pareidolia. Methodological issues such as using control stimuli that are hardly comparable with targets, different task demands, small and/or inhomogeneous samples of participants, different tools as well as data processing strategies may account for controversy in the outcome of brain imaging studies. First of all, here, the face-pareidolia images and control images were rather similar, with the same overall amount of stimulus information available. Previous work routinely used faces and scenes as a control condition for face-pareidolia images. Comparable visual input between Face-n-Thing images either eliciting face pareidolia (when presented upright) or not (when presented inverted) is obviously a prodigious advantage. Second, by contrast with most of the earlier studies, a straightforward task was administered here: On each trial, participants had to indicate whether or not they had a face impression (they were explicitly told that there were no correct or incorrect responses). In other studies, participants had either to respond to inverted images only (32), images of animals only (5), or to specify whether the images were tilted to the right or left (12). In all these cases, it is only assumed that face pareidolia occurred on each single trial entering data processing. Finally, previous work did not examine alterations in gamma oscillations as an indicator of face pareidolia. At this point, it is vital to stress that the present findings nicely dovetail with the outcome of earlier MEG work on face pareidolia that used Arcimboldo-like Face-n-Food images: Early (0 to 0.3 ms) bursts in gamma oscillations were similar to images either triggering or not face pareidolia (8).

### Face Pareidolia and Hemispheric Lateralization.

Similar to the core brain network underlying face processing (44, 45) most of the previous work (not only with Face-n-Thing images used here but also with Arcimboldo-like images and prototype faces containing just a few blobs located in accordance with a coarse face scheme) speaks rather in favor of a predominantly right-hemispheric brain processing [(16, 25–27, 43) but cf. (46)]. For instance, over the right hemisphere, the N170 is larger in response to faces and Face-n-Thing images than to objects (5). The right STS distinguishes faces from Face-n-Thing images (32). In infants aged 20 mo, the EEG response to Face-n-Thing images is magnified in the occipitotemporal areas and becomes right-lateralized in the presence of maternal body odor (9). However, right-hemispheric dominance apparently contradicts lesion data: damage to the right occipitotemporal brain areas leaves face pareidolia in Arcimboldo portraits intact (47–49). At the same time, right-hemispheric brain damaged patients exhibit a higher rating of pleasantness for Arcimboldo portraits than healthy controls (50) presumably because they do not see them as faces, which are often bizarre, grotesque, and sometimes even ugly (2).

The present findings support right-hemispheric dominance of the brain response to Face-n-Thing images, with bursts of gamma oscillations originating primarily from the areas constituting the social brain. However, the right hemisphere is obviously not working alone. The analysis of connectivity unfolding over time indicates an extensive interhemispheric exchange of information occurring throughout all processing stages of face-pareidolia images.

### Dynamic Brain Communication Underwriting Face Pareidolia.

The cutting-edge analysis of brain connectivity unfolding over time (Fig. 4) reveals mutual feedforward and feedback intra- and interhemispheric communication not only within the social brain but also within the extended large-scale network of a number of down- and upstream regions. In particular, the STS and INS are strongly engaged in communication with each other and further brain regions either as signal transmitters or recipients. By contrast, the ITG, whose involvement is revealed also by fMRI (28), seems to be engaged primarily in early brain communication. All these areas are considered as pivots of the social brain (51–56), and especially, the STS appears to be a *top dog* in the face pareidolia network (5, 24, 32). The INS is associated with emotional aspects of face processing (44) that habitually accompany face pareidolia (2, 13, 22). In agreement with this, whole-brain fMRI in awake rhesus macaques shows that the ITG and STS exhibit selectivity for face-pareidolia images as compared to nonface objects (20). The PoG is associated not only with somatosensory communication but also with affective face processing (57) and emotion regulation in general (58). The areas responsible for more general integration of information such as the IPL are also engaged in brain communication underlying face pareidolia, albeit the IPL is involved in emotional face processing too (59).

In a nutshell, the outcome provides a framework for future brain imaging research in patients with mental and neurological conditions characterized by aberrant face pareidolia (and face processing at large) such as schizophrenia (SZ) (6, 10), autism spectrum disorders (ASD) (29, 60, 61), and Parkinson’s disease (62) that are known for male prevalence. Gaining knowledge about the specific patterns of possible alterations in brain communication in these patient populations will provide unique insights into the origins of social cognition deficits.

## Methods

### Participants.

Twenty-two male participants were enrolled in the study. They were aged 36.8 ± 8.6 y (age range, 22 to 49 y). The sample size was determined prior to the study considering possible dropouts. All participants either had normal vision or wore MEG-conform refractive compensation. None had a history of neurological or mental disorders including ASD, SZ, or MDD. These participants served as controls in our previous study on MDD (8). None of them reported regular intake of medication including psychoactive drugs. In order to avoid the influence of cultural differences on the outcome (10), all but one of the participants were German natives. As our previous work (3, 10) showed that impact of display inversion on face pareidolia in Face-n-Thing images is more pronounced in males than in females, we focused on male individuals in order to attain group homogeneity. The other reason for setting a focus on males was some evidence for the considerable sex differences in gamma oscillatory MEG activity on visual-perceptual tasks (63). Yet, for better understanding the brain mechanisms underlying face pareidolia, it is worthwhile in future studies, to take a close look at oscillatory activity and brain communication in female individuals. The study was conducted in line with the Declaration of Helsinki and approved by the local Ethics Committee at the Medical School, Eberhard Karls University of Tübingen. Informed written consent was obtained from all participants. Participation was voluntary, and the data were processed anonymously. Participants received monetary reimbursement for their participation.

### Experimental Design and Procedure.

The task and procedure were similar to those of our previous behavioral studies and described in detail elsewhere (3, 10, 11). We adapted them for the requirements of MEG recording. In brief, participants were administered a computer version of the task by using Presentation software (Neurobehavioral Systems, Inc., Albany, CA, USA). They were presented with a set of nonface face-like Face-n-Thing images (such as houses and waves; Fig. 1) to varying degree resembling a face. Within the MEG chamber, the stimuli were projected onto a screen by means of a PROPixx 1,440 Hz DLP LED Projector (VPixx Technologies Inc., Saint-Bruno, QC, Canada). They subtended a visual angle of 10.2° at an observation distance of 70 cm (image size: 12.5 × 12.5 cm). The images were shown in a pseudorandomized order, one by one for a fixed duration of 1.2 s with either canonical or inverted (rotated to 180° in the image plane, Fig. 1) orientation. In total, each experimental session consisted of 224 stimuli [14 images × 2 types (original/mirror image) × 2 display orientations (upright/inverted) × 4 repetitions]. No more than four images with the same orientation (either upright or inverted) appeared consecutively; in this way, a possible adaptation of the visual system and brain to display orientation was prevented. Prior to each image, a white fixation cross was displayed in the center of the screen for 2 s. By contrast with the most previous neuroimaging work (5, 12, 31), participants were directly required to report face pareidolia: On each trial, in a 2AFC task, participants had to indicate whether or not they had an impression of a face by pressing a respective key. Participants were explicitly told that there were no correct and incorrect responses on the task, and they had to rely solely upon their own visual impression. To prevent interference of MEG traces with motor responses, participants were asked to respond after stimulus offset only. If participants failed to respond, the subsequent trial automatically started after an interstimulus interval jittered from 3 to 5 s. For each participant, the recording session lasted for about 12 to 15 min.

### MEG Acquisition and Analysis.

#### MEG recording.

MEG recording with different images (Face-n-Food) is described in detail elsewhere (8). In brief, a whole-head MEG system with 275 axial first-order magnetic gradiometers (VSM MedTech Inc., Coquitlam, BC, Canada) was used. Neuromagnetic signals were recorded at a rate of 1,171.88 Hz with a 293-Hz antialiasing low-pass filter. Participants were seated in an electromagnetically shielded chamber (Vacuum Schmelze GmbH & Co. KG, Hanau, Germany) at the MEG Center, University of Tübingen Medical School. Their head position was determined throughout each measurement by three localization coils positioned at the nasion, as well as left and right preauricular fiducial sites. To minimize the contamination of brain activity by eye-movement artifacts, participants were asked to blink only between trials, if necessary. MEG data analysis was conducted with in-house MATLAB scripts (version, MATLAB 2020a; The MathWorks Inc., Natick, MA) and the Fieldtrip toolbox [version, fieldtrip-20201229; (64)].

#### Preprocessing, time-frequency representations (TFR), and statistical analysis.

The data were grouped based on Display Orientation (upright/inverted) and Response (face/no-face). A detailed description of data preprocessing as well as TFR analysis is given in *SI Appendix, Supplementary Material*. Statistical analysis of TFR data was performed by means of cluster-based nonparametric permutation tests. These clusters contained spatio-temporo-spectral, i.e., three-dimensional (3D) data. We chose two-tailed cluster-based test statistics with a cluster-α of 0.05 and a required minimum cluster size of two neighboring channels. This method has proven sufficient to correct for family-wise error rates with multiple comparisons (65). The channel-time-frequency triplets with a *t*-value exceeding the cluster-α were grouped on the basis of their spatial, spectral, and temporal adjacency. The Monte Carlo method based on a set of 1,000 permutations was used to calculate the significance level (α = 0.05). Subsequently, the maximum sum of *t*-values was used to construct permutation-based random distributions for two conditions (face/no-face). Statistical testing to infer a possible effect of condition (face/no-face) was then accomplished on relative power changes with respect to baseline (0.5 to 0.3 s prestimulus) for the time window of stimulus presentation (0 to 1.2 s). To obtain a finer frequency resolution, statistical analyses were performed independently for individual frequencies in the gamma range of 30 to 95 Hz in steps of 5 Hz.

#### Source reconstruction.

Source localization based on time windows and frequency ranges of the significant clusters was performed using a beamformer approach implemented in the Fieldtrip toolbox (similar to ref. 8). To obtain cross-spectral density (CSD) data, a TFR analysis was conducted using discrete prolate spheroidal sequence tapers. The spectrum was calculated for the mean of significant clusters’ frequency range with a time window of 0.5 s sliding forward in steps of 0.05 s from 1.25 s pre- to 1.25 s poststimulus. Furthermore, a canonical Montreal Neurological Institute template was specified and segmented in a coordinate system defined by the fiducials nasion, left and right preauricular sites. A single-shell volume conduction model was derived from the brain scan (66). A regularly spaced grid was established with 1-cm spacing in 3D space. Source analysis was based on the dynamical imaging of coherent sources approach (67). Common spatial filters for both conditions were derived from the CSD matrix of the TFR data and leadfield matrix. Applying the spatial common filters resulted in TFRs for each condition and grid point, reflecting the time course of the spectral source strength.

#### Time-based connectivity analysis.

The connectivity analysis was performed for the frequency band of 80 to 85 Hz that exhibited a significant difference in spectral power between conditions. This analysis assumes that increased coherence is indicative of communication between the engaged brain regions. Overall, five regions of interest (ROIs) were chosen based on their maximum power spectrum values of the source localization outcome (all but one of ROIs were found in the right hemisphere): the STS-R, ITG-R, INS-R, PoG-R, and the IPL-L. The same regions were also taken in the opposite hemisphere. In order to assess directionality of the connections (leading or lagging) and their potential changes over time (i.e., time course), the phase slope index (PSI) for the connections of ten ROIs with the other ROIs was calculated in each participant from stimulus onset over the entire stimulus duration in 0.4-s windows with an overlap of 0.1 s (as stimulus duration was 1.2 s, overlap for the last window was 0.2 s). For each connection, mean and SD of PSIs were computed, and the values of individuals deviating more than ±2 SD from the mean (considered outliers; one to three participants per connection) excluded. Finally, a two-tailed sign test was performed to test for the significance of the connection directionality.

#### Normality of data distributions.

All datasets were routinely checked for normality of distribution with the Shapiro–Wilk test. We further used parametric statistics for normally distributed data. For non-normally distributed data, nonparametric statistics was performed, and we reported Mdns and 95% CIs, in addition to means and SDs. Statistical testing of behavioral data was accomplished with the JMP package (version 16; SAS Institute, Cary, NC) and MATLAB (version MATLAB 2020a; The MathWorks Inc., Natick, MA).

## Supplementary Material

Appendix 01 (PDF)

Movie S1.

## Data Availability

All study data are included in the article and/or supporting information.
