# Supplementary material for: Dynamic brain communication underwriting face pareidolia
Source: Proc Natl Acad Sci U S A. 2024 Apr 8;121(16):e2401196121. doi: 10.1073/pnas.2401196121 (PMC11032489; doi:10.1073/pnas.2401196121)
Supplement: Supplementary file 1 — Appendix 01 (PDF) [file pnas.2401196121.sapp.pdf]

Supplementary material

Supplementary Methods

Data preprocessing

For all participants, based on recorded triggers, the individual MEG datasets were automatically divided into 224 trials. Each trial encompassed a time period starting from 1.5 s before through 1.5 s after stimulus onset. This allowed controlling for possible filter artifacts at both ends of single segments. Data were down-sampled to 250 Hz and high-pass filtered at 1 Hz. Employing visual examination, trials characterized by great variance of brain activity across channels ($> 2 \times 10^{-25}$ T^2/Hz) were discarded from subsequent analyses. In ten datasets, channels with large variance across trials were removed with a maximum of 21 channels per participant. The eliminated channels were interpolated for further sensor-level analyses. An independent component analysis (ICA) was conducted for a more elaborated artifact rejection (1, 2). After decomposition of individual traces into 272 components, the first 80 components were visually inspected for muscular, ocular and cardiac artifacts. Contaminated trials/components were removed, and a cleaned MEG signal was generated. In total, 4,369 trials of 22 participants (198.59 ± 22.38 trials per participant) passed all steps of preprocessing.

Time-frequency analysis

After preprocessing, individual trials were grouped based on Display Orientation (upright/inverted) and Response (face/non-face). Face responses with upright display orientation were compared with non-face responses with inverted orientation. In such a way, a maximum possible contrast could be achieved for generation of time-frequency representations, TFRs. For ruling out any effect of unbalanced trial number on TFR, the condition (face responses/non-face responses) with fewer trials (N_{min}) was identified and contrasted with randomly chosen N_{min} trials of the other condition. For each condition and each participant, a

TFR was calculated from 2 to 98 Hz with a frequency resolution of 2 Hz. To minimize spectral leakage of TFR, we used Hanning tapers with a fixed time window of 500 ms. The sliding window was shifted in steps of 50-ms covering a period ranging from 1.25 s before to 1.25 s after stimulus onset (3, 4). As a final step, grand averages of TFR data were calculated across participants for both conditions.

Supplementary Results

Connectivity analysis

The connectivity analysis between the regions with increased gamma activity for images eliciting face pareidolia unfolding over time revealed that from the stimulus onset till 0.4 s, the ITG-R receives messages from the INS-R (mean connection strength as assessed by the PSI, 0.041±0.079 (SD), $p = 0.041$), and sends information to the left IPL-L; 0.051±0.089, $p = 0.003$). In addition, the IPL-L exhibits a tendency to transmit signals to both the INS-L (0.05±0.106, $p = 0.053$) and PoG-L (0.03± 0.068, $p = 0.078$). The STS-R exhibits a tendency to receive information from the ITG-L (0.012±0.077, $p = 0.078$) and transmits signals to the INS-R (0.049±0.106, $p = 0.078$), which transfers information further to the PoG-R (0.031±0.096, $p = 0.078$) of the same hemisphere.

In the time window of 0.3-0.7 s, the STS-R transmits signals to the STS-L (0.049±0.106, $p = 0.027$), which exhibits a tendency to receive information also from the INS-L (0.043±0.096, $p = 0.078$). The INS-R is engaged in active communication both with the left hemisphere transferring information to the ITG-L (0.051±SD 0.081, p = 0.017), and within the right hemisphere exhibiting a tendency of signal transfer to the IPL-R (0.072±0.124, $p = 0.078$) and PoG-R (0.058±0.108, $p = 0.053$).

During 0.6-1.0 s, both the STS-L and INS-L transfer signals to the right-hemispheric brain areas. The STS-L actively communicates with the STS-R (0.071±0.083, $p = 0.012$). The INS-

L communicates with the INS-R (0.045±0.077, $p = 0.041$), IPL-R (0.049±0.070, $p = 0.041$), and less strongly with the PoG-R (0.039±0.072, $p = 0.078$).

By contrast, in the time window of 0.8-1.2 s, both the STS-R and INS-R transfer signals to the PoG-L (0.078±0.081, $p = 0.012$; 0.051±0.081, $p = 0.012$, respectively). Interestingly, not only the STS-R transfers signals to the PoG of the opposite hemisphere (PoG-L), but also the STS-L does so, showing a tendency of signal transfer to the PoG-R (0.037±0.106, $p = 0.078$). Finally, in this late time window, the INS-L receives information from the IPL-L (0.039±0.098, $p = 0.041$).

Supplementary References

1. A. J. Bell, T. J. Sejnowski, An information-maximization approach to blind separation and blind deconvolution. *Neural Comput* **7**, 1129-1159 (1995).
2. S. Amari, T. P. Chen, A. Cichocki, Stability analysis of learning algorithms for blind source separation. *Neural Networks* **10**, 1345-1351 (1997).
3. D. B. Percival, A. P. Walden (1993) Spectral analysis for physical applications. (Cambridge University Press).
4. P. P. Mitra, B. Pesaran, Analysis of dynamic brain imaging data. *Biophysical Journal* **76**, 691-708 (1999).
