# Supplementary figures and images for: Dynamic brain communication underwriting face pareidolia
Source: Proc Natl Acad Sci U S A. 2024 Apr 8;121(16):e2401196121. doi: 10.1073/pnas.2401196121 (PMC11032489; doi:10.1073/pnas.2401196121)

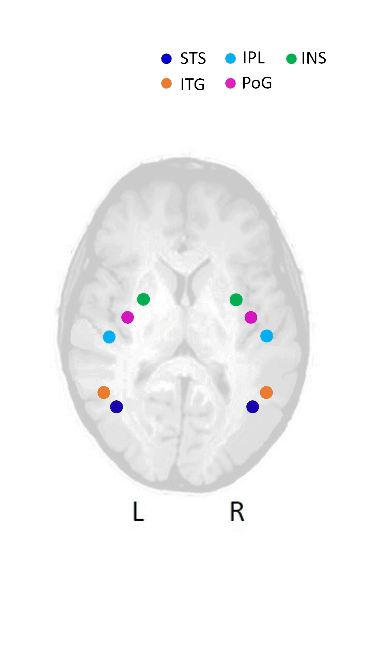

Supplement: Movie S1. [file pnas.2401196121.sm01.gif]
